# Tailoring Functionality of Nanocellulose: Current Status and Critical Challenges

**DOI:** 10.3390/nano13091489

**Published:** 2023-04-27

**Authors:** Yidong Zhang, Wangfang Deng, Meiyan Wu, Mehdi Rahmaninia, Chunlin Xu, Bin Li

**Affiliations:** 1 Laboratory of Natural Materials Technology, Åbo Akademi University, Henrikinkatu 2, FI-20500 Turku, Finland; 2 CAS Key Laboratory of Biofuels, Qingdao Institute of Bioenergy and Bioprocess Technology, Chinese Academy of Sciences, Qingdao 266101, China; 3 Wood and Paper Science and Technology Department, Faculty of Natural Resources, Tarbiat Modares University, Noor 46417-76489, Iran; 4 Shandong Energy Institute, Qingdao 266101, China; 5 Qingdao New Energy Shandong Laboratory, Qingdao 266101, China

**Keywords:** nanocellulose, surface functionality, TEMPO-mediated oxidation, periodate oxidation, esterification, etherification, silanization, surface coating, grafting, potential application

## Abstract

Nanocellulose (NC) isolated from natural cellulose resources, which mainly includes cellulose nanofibril (CNF) and cellulose nanocrystal (CNC), has garnered increased attention in recent decades due to its outstanding physical and chemical properties. Various chemical modifications have been developed with the aim of surface-modifying NC for highly sophisticated applications. This review comprehensively summarizes the chemical modifications applied to NC so far in order to introduce new functionalities to the material, such as silanization, esterification, oxidation, etherification, grafting, coating, and others. The new functionalities obtained through such surface-modification methods include hydrophobicity, conductivity, antibacterial properties, and absorbability. In addition, the incorporation of NC in some functional materials, such as films, wearable sensors, cellulose nanospheres, aerogel, hydrogels, and nanocomposites, is discussed in relation to the tailoring of the functionality of NC. It should be pointed out that some issues need to be addressed during the preparation of NC and NC-based materials, such as the low reactivity of these raw materials, the difficulties involved in their scale-up, and their high energy and water consumption. Over the past decades, some methods have been developed, such as the use of pretreatment methods, the adaptation of low-cost starting raw materials, and the use of environmentally friendly chemicals, which support the practical application of NC and NC-based materials. Overall, it is believed that as a green, sustainable, and renewable nanomaterial, NC is will be suitable for large-scale applications in the future.

## 1. Introduction

**Figure 1:**
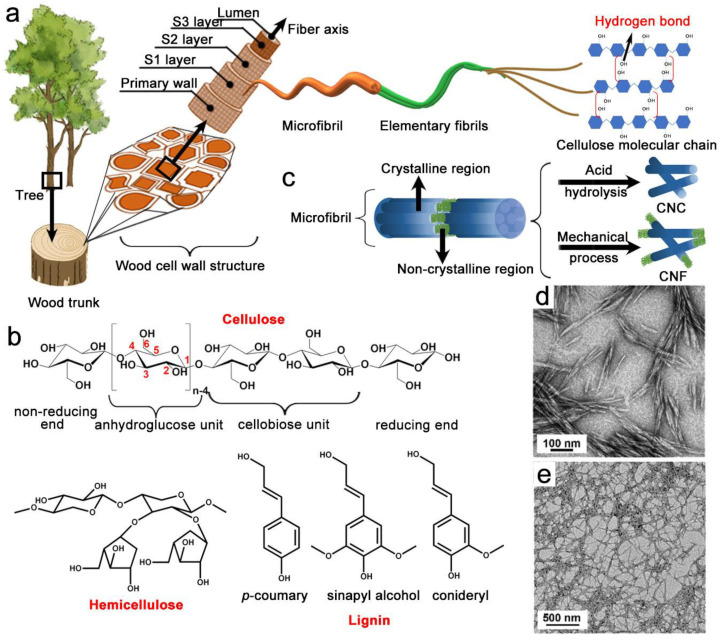
Hierarchical structure of cellulose in plants (**a**); molecular structures of cellulose, hemicellulose, and lignin (the three primary components of the plant cell wall) (**b**); schematic illustration of the preparation of CNC and CNF (**c**); TEM images of CNC (**d**) and CNF (**e**) [21].

Environmental awareness has focused significant attention on the better utilization of sustainable natural polymers, such as cellulose, chitin, and starch [1,2]. Cellulose, which has a polysaccharide structure, is abundantly available across the Earth, and it is present in woody and non-woody plants, as well as some sea animals, such as tunicates [3,4]. Cellulosic fibers were used as lumber, Chinese Xuan paper, textiles, and cordages for thousands of years [5]. The French chemist, Anselme Payen, first isolated cellulose from plants in 1839, and Staudinger later determined the polymeric structure of cellulose in the 1920s [6]. In nature, all plants, even tall trees (some of which are over 120 m in height), are supported by the hierarchical structures of wood-cell walls, which consist of the primary cell, the secondary wall, and the lumens (Figure 1a) [7]. In particular, the secondary wall (thickness ≈ 4 µm) can be further subdivided into three concentric layers, S1–S3, and the lumen forms the hollow center [8]. Furthermore, the S2 layer holds most of the cellulose microfibrils (MFs), with diameters in the range of 10–30 nm and lengths reaching more than 2 µm [9,10,11]. The MFs can be further divided into tens of elementary fibrils (EFs), with diameters ranging from 3 to 5 nm and lengths reaching more than 1 µm [12]. Furthermore, the fact that EFs are surrounded by hemicellulose and lignin and hierarchically packed together endows MFs with extraordinary structural stability. The EFs are formed from cellulose-molecule chains, which are considered fundamental parts of the wood cell wall. Each cellulose molecule has abundant hydroxyl (–OH) groups that can form intra-hydrogen bonds, thus stabilizing the nanofibers through inter-hydrogen bonds and promoting parallel chain stacking [13]. Hemicellulose constitutes 20–30% of the wood dry mass. Compared to cellulose, hemicellulose has a shorter crosslinked chain structure (DP ≈ 200) (Figure 1b) [14]. Additionally, hemicellulose is made of several monomers (e.g., glucose, xylose, and arabinose), whereas cellulose is comprised of glucose monomers only [15]. Moreover, compared to cellulose, the remarkable aspect of hemicellulose is its diversity in terms of the types of side group, the units of composition, the molecular weights, and the branching sites [16]. The diversity of hemicellulose present challenges when examining the relationship between its intrinsic heterogeneity and the properties of its final products. Furthermore, hemicellulose combines with cellulose, via hydrogen bonds, and lignin, via hydrogen bonds and ether bonds, strengthening the stability of the cell wall [17]. Lignin is a complex phenolic polymer of aromatic compounds with three main aromatic subunits (*p*-coumaryl alcohol, coniferyl alcohol, and sinapyl alcohol), which are mainly linked together by C-C bonds and ether bonds, leading to a complex and irregular macromolecular structure (Figure 1b) [18,19]. Lignin is hydrophobic by nature, and has a much higher degree of crosslinking than hemicellulose, which contributes to the elasticity and mechanical strength of the plant [20].

Nickerson and Habrl [22] extracted nanomaterials named nanocellulose (NC) from cotton linters by using sulfuric-acid hydrolysis in 1947. Since then, various physical and chemical properties of NC, such as its low weight, low cost, high strength, stiffness, and non-toxic properties have been comprehensively investigated in both academia and industry. Due to the presence of abundant surface hydroxyl groups, NC can be easily functionalized by amination, silanization, carboxylation, or esterification to obtain different cellulose derivatives. Moreover, some small molecular substances, such as dopamine, tannic acid, acrylate, and acrylamide, can be coated or grafted on the surface of cellulose to obtain functional NC. The functionality of NC is of vital importance for its final applications. However, there is a comprehensive summary focused on the introduction and definition of NC substrates, the chemical modification routes applied so far for the functionalization of NC, and NC-based functional materials is still lacking. Moreover, it still remains a challenge to modify NC simply and efficiently without destroying the original morphology and crystalline structure. Herein, this review summarizes the conceptual methods applied to, the current status of chemical modification routes for, and the morphologies and reactions of different functionalized NCs, including TEMPO-mediated oxidation, periodate oxidation, esterification, etherification, silanization, surface coating, and grafting. Most importantly, the functionality of NC is strongly linked to its application performance. Therefore, we correspondingly introduce NC-based functional materials, such as paper-based devices, antimicrobial packaging, pollutant absorption, conducting polymer hydrogels, wearable sensor, and flexible electrodes. In short, unlike other reviews, this review paper mainly summarizes the up-to-date functionalization modification of NC and NC-based functional materials.

## 2. Structures and Characteristics of Cellulose and Nanocellulose

### 2.1. Cellulose

Driven by the awareness of the better utilization of sustainable natural polymers, cellulose could serve as a promising alternative material for petroleum-based materials due to its virtues of abundance, sustainability, degradability, and biocompatibility [1]. Cellulose is abundantly available from plants and other sources, such as trees, bamboo, hemp, cotton, agricultural crops, bacteria, tunicates, and algae [23,24,25]. Moreover, as a green polymer, cellulose is suitable for utilization in sustainable materials engineering. From a top-down perspective, lignin and hemicellulose need to be dissolved/depolymerized from plants, followed by processing to obtain cellulose for various end uses [26]. Cellulose with repeated cellobiose units is the most abundant natural polysaccharide. As shown in Figure 1b, the cellobiose unit is assembled by two anhydroglucose rings rotated by 180° relative to each other and connected by β-1,4 glycosidic bond [24]. The general formula of cellulose is (C_6_H_10_O_5_)_n_, where n is the degree of polymerization (DP), depending on the cellulose’s source material and the preparation approach [14]. Each cellulose chain has a hemiacetal group and chemically reducing functionality, and the other end possesses a pendant hydroxyl group, the nominal non-reducing end.

Moreover, bacterial cellulose (BC, a kind of microbial cellulose), with high levels of water retention and a specific surface area, is typically synthesized by Gram-negative or Gram-positive bacteria (including *Acetobacter xylinum*, *Acetobacter*, *Alcaligenes*, *Pseudomonas*, and others) [27,28]. The DP of BC varies from 2000 to 6000, with diameters in the range of 10–50 nm and lengths in the range of 100–1000 nm [29,30]. Bacterial cellulose has been widely used in food additives, bio-medical sectors, and bio-based nanocomposites due to its high purity, distinct physicochemical characteristics, and biodegradability [31]. Different forms of BC can be produced by changing its mode of fermentation. Under agitation or stirring, sharp or irregular sphere-like BC particles are formed, while BC materials with three-dimensional interconnected structures can be produced in static conditions.

### 2.2. Nanocellulose

As is widely known, various chemical pulping and bleaching reactions are adopted to fully remove lignin and partially remove hemicelluloses to obtain cellulose pulp [7]. Next, mechanical treatments (e.g., high-pressure homogenization, high-intensity ultrasonication, or ultrafine grinding) or inorganic acid hydrolysis (e.g., sulfuric, hydrochloric, maleic, or phosphoric acid) are used to refine the cellulose pulp (Figure 1c) [32,33,34,35,36]. Subsequently, NC derived from lignocellulose is divided into two generic forms, cellulose nanofibril (CNF) and cellulose nanocrystal (CNC) [37]. In general, CNF is a flexible, fiber-like, and semicrystalline cellulose nanomaterial with diameters of less than 100 nm, typically ranging from 3 to 50 nm, and lengths reaching over 1000 nm (Figure 1d) [38]. Cellulose nanocrystal is a rigid rod-like cellulose nanomaterial with a diameter of 10–30 nm and a length of 5–200 nm, which is mostly crystalline in nature (Figure 1e) [39]. The properties (length, diameter, aspect ratio, modulus, strength, and specific surface area) of NC generally depend on the cellulose source, as well as the preparation method and conditions (such as the concentration of chemicals, the treatment temperature, and the treatment time).

It is possible to produce CNFs with a high aspect ratio (>100), excellent tensile modulus, and entangled morphologies, by using mechanical nano-fibrillation methods such as homogenization, ultrasonication, microfluidization, and grinding. In particular, high-pressure homogenization is the most commonly used method for producing CNFs with high quality, which can break down cellulose-pulp fibers and release nanofibrils through various forces, such as rapid changes in high pressure, strong shear, high speed, and turbulence [40,41]. However, some issues need to be carefully addressed when using this method, particularly its large energy consumption and relatively low production yield. Thus, some pretreatments were invented recently to address these drawbacks, such as 2,2,6,6-tetramethylpiperidine-1-oxyl (TEMPO)-mediated oxidation, formic acid (FA) hydrolysis, ionic liquid (IL) treatment, enzymatic hydrolysis, carboxymethylation, and others [42,43]. These pretreatments contribute to the swelling of the fiber wall, which effectively loosens the interfibrillar hydrogen bonds, which supports the subsequent mechanical treatment. It should be pointed out that some pretreatment methods (such as TEMPO-mediated oxidation and FA hydrolysis) do not only reduce the energy consumption of the subsequent mechanical homogenization during NC production, but also simultaneously introduce some new functional groups (carboxyl and ester groups, accordingly) into NCs during pretreatment, which endow the NCs with different surface properties, which are heavily linked to its application performance. Enzyme pretreatments, such as cellulase, hemicellulases, and ligninases, have been used to facilitate the production of CNF, which breaks down targeted bonds in pulp [44]. However, the efficiency and activity of enzymatic pretreatments depend on the enzyme dosage, reaction time, pH, and reaction temperature, which limit its application in the large-scale production of CNF. Additionally, green solvents (e.g., ILs, DES) have been extensively used in pretreatments in NC fibrillation because of their ability to loosen the cellulose network by disrupting hydrogen bonds [45].

Unlike CNF, CNC, which has a relatively low aspect ratio, is typically obtained through the strong inorganic acid hydrolysis of cellulosic pulp, which is used to remove disordered and paracrystalline regions in cellulose, such as sulfuric acid, phosphoric acid, hydrochloric acid, nitric acid, and hydrobromic acid [35,46,47]. Among these hydrolyzed acids, sulfuric acid has been widely used for the production of CNC, since CNC demonstrates exceptional dispersity in water, high hydrolytic efficiency, and simple and time-saving process [48]. Generally, the disordered regions (amorphous structure) are mostly degraded during acid hydrolysis due to the loose structure, while the crystalline structure of cellulose remains as CNC due to its stability [49]. However, the issues in the use of sulfuric acid for CNC production, such as the large usage of water, the harsh corrosion of the equipment used, and the relatively low production yield, need to be rigorously addressed [50]. Thus, some recoverable organic acids and solid acids, such as oxalic acid, maleic acid, FA, and phosphotungstic acid, have been used to prepare CNC through a sustainable and environmentally friendly process. For instance, oxalic acid was used to hydrolyze hardwood pulp to prepare CNC, and the highest yield of the CNC was around 25 wt% [51]. Furthermore, the incompletely hydrolyzed solid cellulosic residue was used as feedstock to produce CNF through homogenization. In recent years, FA, as a recyclable organic carboxylic acid, has been used to hydrolyze cellulose for producing CNC [21]. Compared with other inorganic strong acids, FA can be easily recovered and reused due to its lower boiling point (100.8 °C), which is less corrosive to equipment [52]. Additionally, FA can efficiently hydrolyze hemicellulose, remove lignin, and maintain cellulose; thus FA has been widely used to pretreat various cellulosic materials in the production of CNC.

**Figure 2:**
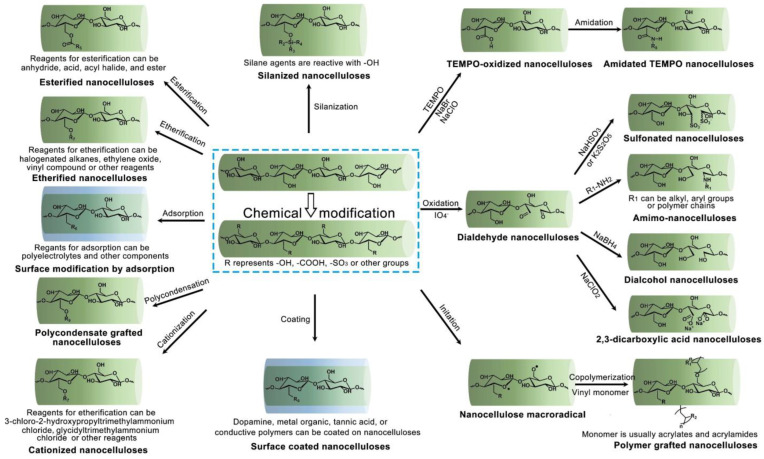
An overview of the tailoring functionality of NC.

The modification of NC is of crucial importance to ensure the functionality of its various final applications. In general, chemical modifications, such as silanization, esterification, and etherification, occur in reaction of different chemicals with the hydroxyl groups of NC. In contrast, some chemical modifiers, such as dopamine, tannic acid, acrylates, and acrylamides, can also be coated/grafted on the surfaces of cellulose materials. These reactions are sensitive to water and are typically present at low concentrations, which increase the reagent consumption and make the whole process environmentally unfriendly and difficult to scale up. Various modification processes have been applied because different NCs have different features. For these reasons, it remains challenging to modify NC surfaces simply and efficiently. Herein, we focus on the chemical modification of NC, and the main surface chemistries of NC mentioned in this paper are presented in Figure 2.

## 3. Tailoring Functionality of NC

As mentioned above, the chemical modification of NC is of great interest in both academia and industry. In this section, we focused on recent chemical modifications of NC, including oxidation, coupling, grafting, surface coating, and others.

### 3.1. TEMPO-Mediated Oxidation of NC

As is known, due to the presence of surface hydroxyl groups, NC can be oxidized by different kinds of oxidizer (e.g., TEMPO, hydrogen peroxide, and periodic acid) to fabricate various cellulose derivatives and introduce many active groups (e.g., carboxyl, aldehyde, and carbonyl functional groups).

**Figure 3:**
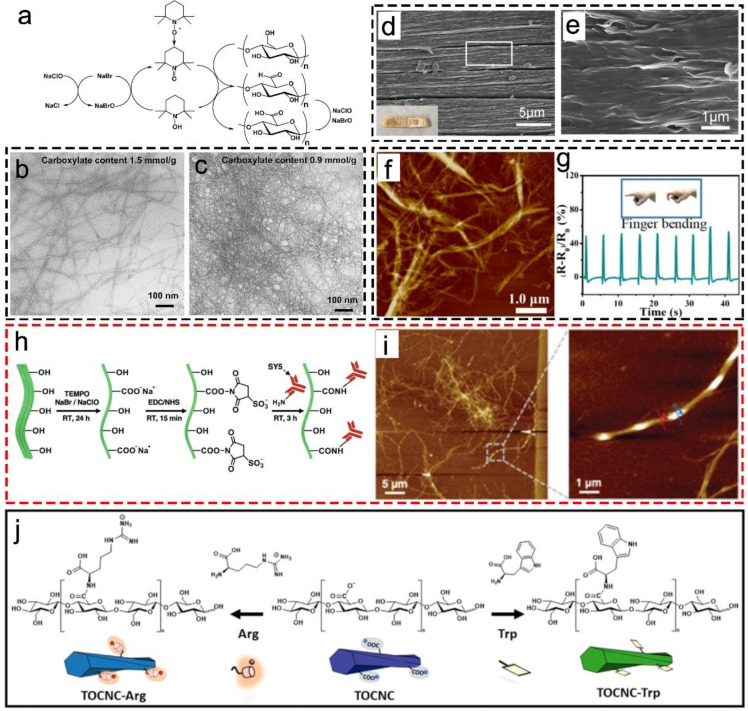
Mechanisms of TEMPO oxidation (**a**) [53]; TEM images of the TOCNF with carboxylate contents of 1.5 (**b**) and 0.9 mmol/g (**c**) [54]; SEM image of the cross-section of the pressed CNF bulk material (**d**,**e**) [55]; AFM image of dialdehyde TEMPO-oxidized NC (**f**) [56]; the recorded relative resistance changes of the strain in forefinger bending (prepared using dialdehyde TEMPO-oxidized NC) (**g**) [56]; schematic illustration of fabrication of antibody protein (SY5)-conjugated TOCNF (**h**), and the corresponding AFM images (**i**) [57]; schematic illustration of fabrication of pendant arginine (Arg) and tryptophan (Trp) into the TOCNC by amide coupling (**j**) [58].

The TEMPO oxidation of cellulose is a kind of pretreatment to isolate NC and obtain CNF or CNC with carboxyl groups (i.e., TOCNF or TOCNC). This reaction selectively replaces OH groups at C6 with aldehydes, after which the aldehyde groups are further oxidized into carboxylic groups at C6 of the glucose unit. This process can be carried out through the TEMPO/NaBr/NaClO reactant system [43]. In general, the oxidation process is conducted using TEMPO with NaClO₂ or NaClO as a secondary oxidant to recycle the TEMPO at pH 9–11. Furthermore, NaBr is the most commonly used reagent to increase the rate of oxidation through the formation of NaBrO in situ. The mechanism underlying the whole process is illustrated in Figure 3a.

As shown in Figure 3b,c, the TOCNF with low carboxylate content (0.9 mmol/g) formed lateral aggregates, and the TOCNF with the carboxylate content of 1.5 mmol/g was mostly converted to homogeneous cellulose fibrils with high average aspect ratios of >150 [54]. In accordance with a previous report, TOCNF-lignin bulk material was prepared [55], and this material exhibited excellent flexural strength (~198 MPa), an extremely light weight (~1.35 g/cm^3^), and good toughness (~8.5 kJ/cm^2^) (Figure 3d,e). Furthermore, TEMPO oxidation is now also used as a pretreatment to further functionalize the surface groups of NC. A dialdehyde TEMPO-oxidized NC was prepared through a TEMPO-oxidation pretreatment followed by NaIO_4_ oxidation [56], and these NCs were used to pre-reinforce gelatin nanocomposite hydrogel (Figure 3f). As the wearable assembly sensor, this hydrogel recorded the relative resistance changes in the strain in forefinger bending with good strain sensitivity and compressive sensitivity (Figure 3g). Furthermore, a TEMPO-oxidized NC was used to prepare hydrogels and films using a conjugated antibody protein or amino acid moieties for a variety of biomedical applications. It was reported that an antibody protein (SY5) was conjugated with TOCNF to produce antigen–antibody interaction with involucrin, which improved wound healing by essentially providing a tissue environment [57]. A schematic illustration of the fabrication of the antibody protein (SY5)-conjugated TOCNF and the corresponding AFM images are presented in Figure 3h,i. As shown in Figure 3i, SY5 with a size of 10 nm was generated along the TOCNF; this was caused by the coupling reaction between the SY5 and the TOCNF. Moreover, cellulose films were fabricated by grafting pendant arginine (Arg) and tryptophan (Trp) onto cellulose, which could be used to control the mechanical properties of cellulose films (Figure 3j) [58].

In addition, the properties of the resultant TOCNF are also dependent on the sources of the raw materials. Usually, wood fiber (cellulose I) is TEMPO-oxidized to obtain surface-modified TOCNF, whereas mercerized and regenerated cellulose (cellulose II and amorphous) are oxidized to obtain water-soluble salt. Saito et al. [59] used bleached sulfite cotton, bacterial cellulose, tunicin, and wood pulp to produce NC. It was found that the restrictive degree of oxidation was reduced in the following order: wood pulp > cotton pulp > tunicin and bacterial cellulose. Although the TEMPO oxidation of cellulose is a simple pretreatment to produce TOCNF, certain issues, such as the long duration of the reaction (1–3 days), severe environmental pollution, and large water usage need to be addressed in the future. Thus, various approaches have been developed to partially replace TEMPO-oxidized chemicals, such as periodate [60], periodate-chlorite oxidation [61], nitric-acid/sodium-nitrite oxidation [62], N-hydroxyphthalimide (NHPI) [63], and ammonium persulfate (APS) oxidation [64]. Among these oxidation methods, APS oxidation is cheaper than TEMPO oxidation for the production of NC at the same oxidation degree. The oxidation ability of APS is attributed to the activated S_2_O_8_^2−^, which can destruct amorphous regions of cellulose. It should be noted that although APS oxidation can be a strong alternative to other oxidative methods for CNF production, it produces CNC instead of CNF, which may limit its application in some respects. On the other hand, compared to TEMPO oxidation, NHPI-oxidizing systems can produce higher contents of carboxylic groups and preserve the morphologies of original samples without the further depolymerization or degradation of cellulose. Additionally, NHPI combined with NaIO_4_ was used to produce highly water-soluble 2,3,6-tricarboxy cellulose, eliminating the water-soluble limitation of cellulose caused by the highly ordered hydrogen-bond network and high crystallinity, which can be used in many practical and scalable purposes [65].

### 3.2. NaIO_4_ Oxidation of NC

**Figure 4:**
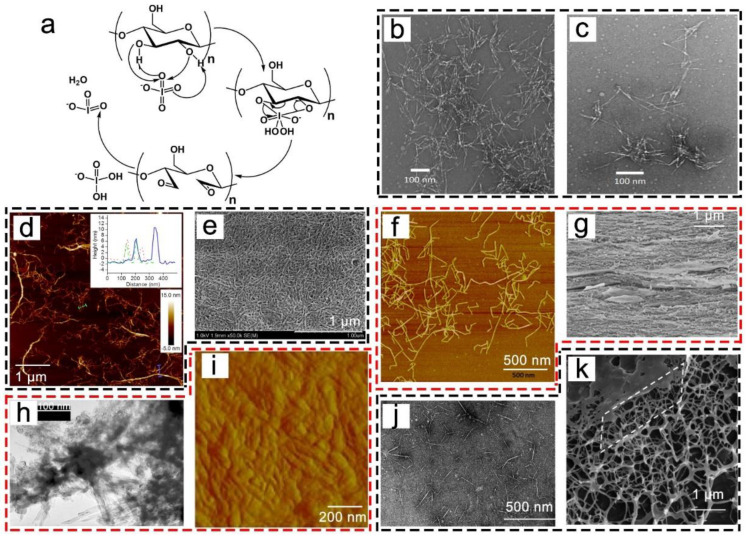
Mechanism of periodate oxidation of cyclic 2,3-dialdehyde (**a**) [6]; TEM images of DANC produced by periodate oxidation for 42 h (**b**) and 84 h (**c**) [67]; AFM image of dialcohol NC (**d**) and SEM images of the fabricated films (**e**) [68]; AFM image of 2,3-dicarboxylic acid cellulose (**f**) and SEM images of the fabricated film (**g**) [69,70]; TEM image of sulfonated cellulose (**h**) and AFM images of the fabricated cellulose nanosphere (**i**) [71]; TEM image of amidated cellulose (**j**) and SEM image of the fabricated hydrogel (**k**) [72,73].

Among the modified NC samples, dialdehyde NC (DANC) can be prepared through selective NaIO_4_ oxidation, which breaks the C2–C3 bond in the glucose repeat units of cellulose and introduces two aldehyde groups per glucose unit (Figure 4a) [66]. Moreover, cellulose can be pretreated by alkali, ultrasound, or molten-salt hydrates, and then oxidized to improve the reaction efficiency. As shown in Figure 4b,c, the DANC produced by periodate oxidation (for 42 and 84 h, respectively) consisted of rigid and straight rods with similar diameters, ranging from 5 to 10 nm [67]. However, their lengths decreased from 240 to 100 nm at 42 h and DANC 84 h, respectively. These results indicated that the morphology of DANC can be effectively controlled by the amount of periodate and the reaction time.

Furthermore, DANC, which contains multiple aldehyde groups, can be further oxidized, sulfonated, or reacted with chemicals with amino groups to produce cellulose-based materials for different applications. Larsson et al. [68] used periodate oxidation and sodium borohydride (NaBH_4_) reduction to produce dialcohol NCs 4–10 nm in diameter and 0.5–2 μm in length (Figure 4d). Subsequently, a strong and tough film with a breaking strength of 175 MPa and a breaking strain of 15% was fabricated (Figure 4e). Furthermore, the dialdehyde groups created by periodate oxidation were converted to carboxylic acid using sodium chlorite (NaClO_2_) to form 2,3-dicarboxylic acid NC [69,70,74,75]. As shown in Figure 4f, dicarboxylic acid NCs ranging from 3 to 5 nm were prepared, while their lengths reached the micrometer-scale [70]. Next, a film was fabricated with tensile strength and Young’s modulus values up to 211 ± 3 MPa and 12 ± 1 GPa, respectively (Figure 4g). Because of the well-organized structures of the hybrids and small pore sizes, the as-fabricated film possessed good oxygen-barrier properties [69]. In addition, periodate oxidation is also an attractive route for the introduction of sulfonated groups into cellulose fibers, which takes place through the addition of potassium persulfate (K_2_S_2_O_8_) or NaHSO_3_ [71,76,77]. The TEM analysis indicated the rod-like aggregates of the sulfonated NC (SCNF, Figure 4h), and the AFM image further exhibited the surfaces of the cellulose nanospheres obtained from the SCNF (Figure 4i). It was found that the water absorbency of the cellulose nanospheres was improved from 8% to 199% with this oxidation/sulfonation protocol [71]. Additionally, NC oxidation by periodate has been used to introduce amine groups using benzylamine [72,73]. Methylamine was used to adjust the hydrophobicity of the NCs, and the obtained NCs exhibited lengths of 73–131 nm and widths of 5–6 nm (Figure 4j) [73]. Moreover, NCs with high hydrophobicity were prepared using octyl moieties to improve the hydrophobic interactions, and the modified NC resulted in stiffer and stronger hydrogels compared to hydrogels reinforced with hydrophilic NCs (Figure 4k) [72].

Periodate oxidation followed by reduction, sulfonation, or oxidation was recently applied for the introduction of functional groups into cellulose fibers. However, these processes are time-consuming and use toxic products, which make them unsustainable and environmentally unfriendly. Thus, further studies are needed to develop a greener process to produce homogenous and functional NC.

### 3.3. Esterification of Cellulose and NC

**Figure 5:**
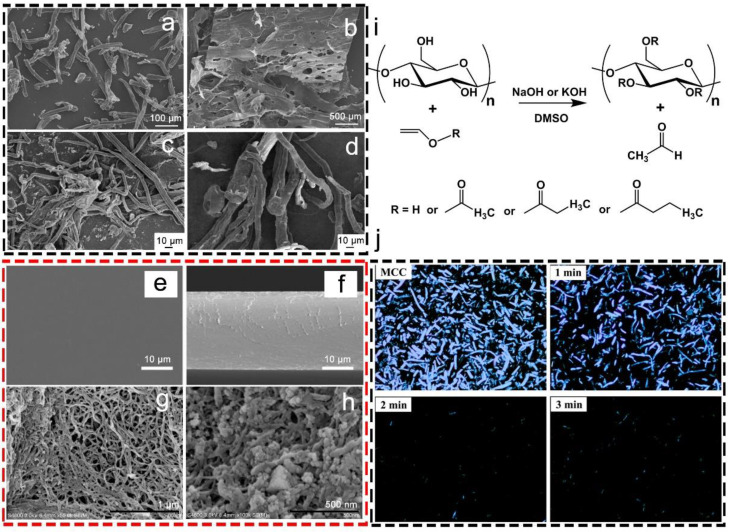
SEM images of alpha-cellulose (**a**) and cellulose sulfate (**b**) [81]; SEM images of miscanthus cellulose (**c**) and cellulose nitrate (**d**) [84]; SEM images of the surface (**e**) and fracture (**f**) regions of the cellulose-acetate films [86]; SEM images of cellulose formate (**g**) and antibacterial cellulose formate (**h**) [87]; transesterification of vinyl esters under the catalysis of NaOH or KOH in DMSO (**i**) [88]; polarizing images of the reaction mixture at different times (DMSO, NaOH, 6:1 molar ratio of vinyl acetate/anhydroglucose unit, 100 °C) (**j**) [88].

As is widely known, there has been extensive research in the esterification of cellulose, which began about 140 years ago. Cellulose esterification can usually be classified as inorganic (e.g., cellulose nitrate, cellulose sulfate, and cellulose phosphate) and organic (e.g., carboxylate esters, transesterification), and it can be used to modify both cellulose and NC [4]. Among these esterification methods, both cellulose nitrate and cellulose sulfate are produced through the esterified hydrolysis of cellulose, and this is followed by homogenization to produce NC [78,79]. Cellulose sulfate was made through the direct esterification of cellulose using sulfuric acid in 1947, by introducing sulfate-half-ester groups onto the surface of cellulose. Subsequently, various methods, including sulfur trioxide, chlorosulfonic acid, or sulfuryl chloride, were widely used for the production of cellulose sulfates [80]. Cellulose sulfates with DS between 1 and 2.6 were prepared through the direct sulfation of cellulose, which exhibited excellent dispersity in water [81]. After the sulfation, the cellulose sulfates, with compact, smooth, and porous surfaces, exhibited completely different morphologies from the cellulose (Figure 5a,b). It was found that the molecular weight of the cellulose was remarkably decreased during the sulfation, which was attributed to the amount of sulfating agent and the sulfation temperature. Cellulose nitrate is one of the most important inorganic cellulose esters, and it can be used in many application fields, including plastics, explosives and coatings [82]. Cellulose nitrate is synthesized by the reaction between cellulose and the classical nitrating acid mixture (nitric acid and sulfuric acid) or nitrating-agent systems (nitric acid/acetic acid/acetic anhydride) [83]. It was reported that miscanthus cellulose was nitrated using an industrial sulfuric-nitric acid mixture to produce cellulose nitrate (Figure 5c,d) [84]. The miscanthus cellulose exhibited a curved and heterogeneous ribbonlike surface, which changed to a three-dimensional fibrous texture after the nitration.

Generally, native cellulose was employed as a starting material to obtain cellulose sulfate, which led to nonuniformly distributed substitution, leading to poor solvability in water [4]. To overcome this limitation, partially modified cellulose derivatives can be used to fabricate cellulose sulfate by means of the displacement of an ester or ether group already present in the cellulose [4]. Furthermore, cellulose sulfates with various substitution patterns can be realized via this method. Carboxyl cellulose sulfates with both sulfate and carboxyl groups were obtained by two synthesis routes [85]. In one, cellulose was sulfated to produce cellulose sulfate, followed by TEMPO-mediated oxidation. In the other, the cellulose underwent TEMPO-mediated oxidation, followed by acetosulfation.

The introduction of organic functional groups onto cellulose via esterification efficiently allows the fabrication of a wide range of valuable products. Cellulose acetate, a typical example of a cellulose-carboxylate ester, is prepared by the reaction between cellulose and the mixture of acetic acid and acetic anhydride, with sulfuric acid as the catalyst [83]. As shown in Figure 5e,f, a cellulose-acetate film was fabricated with glycerol as a plasticizer and n-propanol as a transparent agent [86]. The film exhibited a high transmittance value and a low haze degree of 93.75% and 1.42%, respectively. Furthermore, the film presented a compact structure and a smooth surface, leading to an excellent water-vapor-barrier property (Figure 5f). Additionally, another cellulose ester, cellulose formate (CF), can be fabricated through the esterification between cellulose and FA via a clean and sustainable process (Figure 5g) [87]. The FA is easy to recycle in comparison with conventional inorganic acid, and it can hydrolyze cellulose and react with hydroxyls on the surface of cellulose, thus introducing ester groups (formyl groups) onto the cellulose, generating CF. It was reported that three kinds of nanosized CF with distinct properties were prepared by the one-pot FA hydrolysis of wood pulp, and the formyl groups exhibited exceptional compatibility in a polylactic acid matrix. In addition, it was reported that CF was partly coated by Ag nanoparticles to obtain antibacterial CF, which was caused by a silver-mirror reaction between the CF and silver ammonia (Figure 5h). As expected, the fabricated CF/Ag composites presented strong antibacterial activities against both *Escherichia coli* and *Bacillus subtilis*. Moreover, cellulose esters can be prepared by transesterification in the presence of catalysts. Cao et al. [88] reported an efficient reaction system containing dimethyl sulfoxide (DMSO)/aqueous NaOH or KOH for synthesizing cellulose esters (Figure 5i). The cellulose reacted with vinyl acetate, vinyl propionate, or vinyl butyrate, producing cellulose acetate, cellulose propionate, or cellulose butyrate with a high DS (2.14–2.34) in 5 min. As shown in Figure 5j, the cellulose esters dissolved into the solvent and formed a homogeneous phase within minutes, which was in sharp contrast to previous methods.

On the other hand, the esterification of NC has been widely investigated for further selective applications. It was reported that a functionalized CNCs with a thiol group (CNC-SH) was fabricated by simple and mild organocatalytic esterification, and they were used as sensors by attaching organic catalysts and fluorescent molecules to the surface of CNC-SH [89]. Moreover, chlorotoxin was attached to the surface of NC through Fisher esterification, exhibiting excellent properties in terms of biocompatibility and internalization the in cell line [90]. Furthermore, tetrakis (hydroxymethyl) phosphonium chloride (THPC) was grafted onto the surface of TOCNF via an esterification reaction to prepare THPC@TOCNF membranes, which presented high permeate flux and antibacterial properties [91].

### 3.4. Etherification of Cellulose and NC

**Figure 6:**
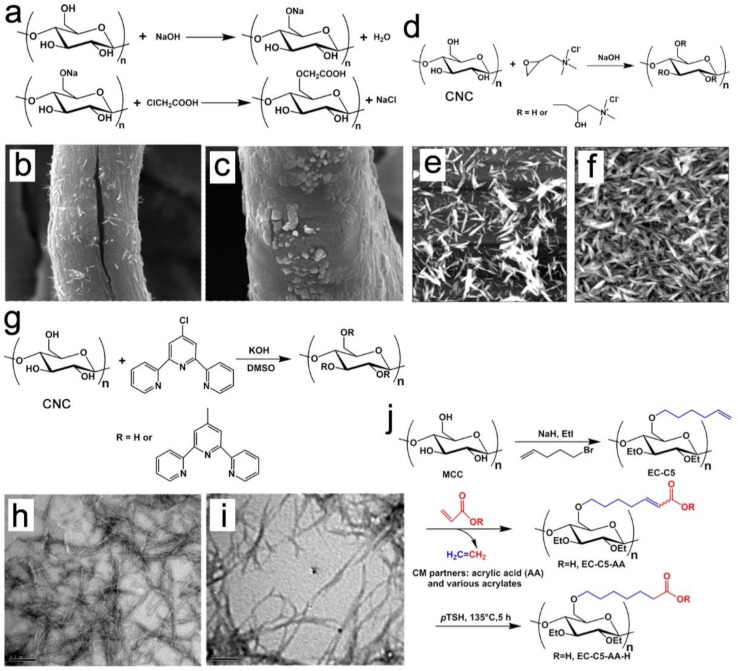
Mechanism of preparation of CMC (**a**); SEM images of ultrasound-microwave-prepared CMC (**b**) and microwave-prepared CMC (**c**) [94]; reaction scheme for surface cationization of CNC with EPTMAC (**d**) [95]; AFM images of CNC before (**e**) and after (**f**) functionalization with EPTMAC [95]; generation of terpyridine-modified NC (**g**) [96]; TEM of non-modified NC (**h**) and terpyridine-modified NC (**i**) [96]; the synthetic of amphiphilic cellulose ether (**j**) [96].

Cellulose ether is an extensively used cellulose derivative, which has been applied in the fields of textiles, membrane science, biological materials, and environmental protection [92,93]. The carboxymethylation of cellulose fibers is a widely used approach to etherification for the preparation of cellulose ether, which can also be used as an efficient method to prepare etherified NC [94]. Sodium carboxymethyl cellulose is produced by converting partially or totally hydroxyl groups with monochloroacetic acid or its sodium salt to carboxymethyl moieties (Figure 6a). Nevertheless, this process presents many drawbacks, such as the toxic halocarbon reactant, high consumption of water, and harsh alkali hydroxide. In order to reduce the chemical consumption, reaction time, and energy consumption, the ultrasound-mediated production of carboxymethyl cellulose (CMC) under microwave irradiation can be carried out. As shown in Figure 6b, cracks on the surfaces of the fibers caused by ultrasonic pretreatment facilitated the penetration of sodium hydroxide and the high reactivity of the cellulose [94]. However, the reaction of the cellulose without sonication was superficial, and most of the reagents were unreacted and remained on the surfaces of fibers (Figure 6c).

Etherification was also used to introduce cationic charges on the surfaces of CNCs (Figure 6d) [6]. The cationization of NC is commonly performed using epoxypropyltrimethylammonium chloride (EPTMAC) in a quaternization reaction [95]. As shown in Figure 6f, due to the cationic nature of the functionalized NC, the EPTMAC-modified NC presented a much more uniform dispersion than the unmodified NC (Figure 6e). Furthermore, surface cationization can generate the stable aqueous suspension of NC through the conversion of hydroxyl groups into an epoxy moiety of EPTMAC. Unfortunately, the hydrolysis of EPTMAC and the presence of by-products in the reaction mixture need to be addressed.

Moreover, highly fluorescent NCs were prepared through the surface modification of terpyridine-modified NCs with terpyridine-modified perylene (Figure 6g) [96]. The terpyridine-modified NCs (Figure 6i) exhibited a slight increase in width in comparison with the unmodified NCs (Figure 6h), while there was no significant change in their lengths. Finally, amphiphilic cellulose ether has also been prepared using 5-bromo-pent-1-ene to produce ethyl pent-4-enyl cellulose, followed by an olefin-cross-metathesis reaction using acrylic acid or acrylate monomer [96]. The steps in the synthesis of the amphiphilic cellulose ether are shown in Figure 6j. The as-fabricated amphiphilic cellulose ether can be used in the fields of drug delivery and waterborne coatings.

### 3.5. Silanization of NC

**Figure 7:**
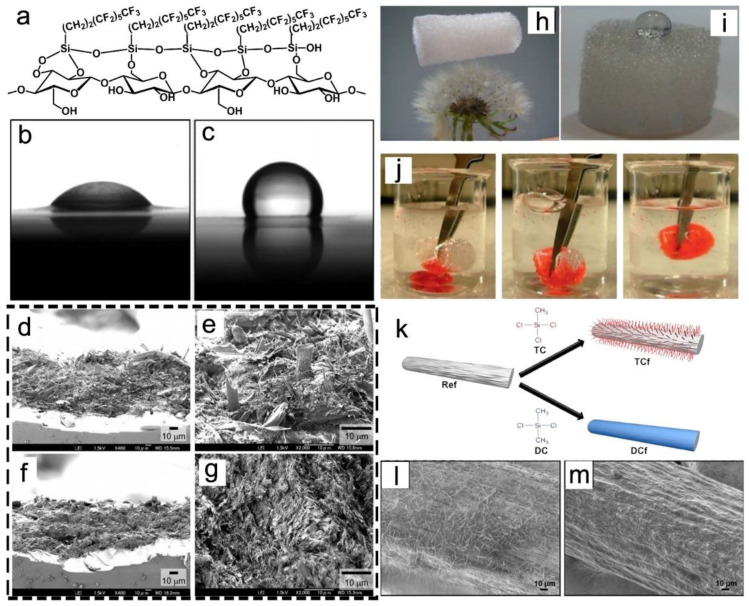
(**a**) Chemical structure of PFOTES-treated NC (**a**) [97]; water-contact angles of the NC films (**b**) and PFOTES-treated NC films (**c**) [97]; SEM images of fractured surfaces of untreated (**d**,**e**) and APS-treated (**f**,**g**) NC composites [98]; photograph of an aerogel on top of a dandelion (**h**) [99]; photograph of a water droplet on top (**i**) [99]; sequential snapshots of removal of Sudan IV dyed chloroform at the bottom of water (**j**) [99]; schematic illustration of the chemical modification of a CNF filament with trichloromethylsilane (TC) and dimethyldichlorosilane (DC) (**k**) [100]; SEM images of TC (**l**) and DC (**m**) treated CNF filaments [100].

The silanization of NC is very important to increase the hydrophobic property of NC, which is applied to packaging, membranes, or specialty paper [97]. It was reported that silanization-modified NC films with excellent tensile strength and hydrophobic properties were prepared through the vacuum filtration of the CNF suspension followed by immersion in perfluoroctyltriethoxysilane (PFOTES) solution (Figure 7a) [97]. The water-contact angle of the untreated NC films was lower than 25°, while the contact angle of the PFOTES-treated NC films increased up to 130.2° (Figure 7b,c). In 2015, Ifuku and Yano treated the surfaces of NC sheets with a silane coupling reagent (γ-aminopropyltriethoxysilane (APS)) to improve the fiber–matrix adhesion [98]. Subsequently, the silane-treated sheets were impregnated with neat acrylic resin followed by hot pressure. The untreated cellulose fibers were observed on the fractured surface, and the surfaces of the fibers appeared clean without sticking to the matrix, which was caused by the low compatibility between the fibers and the resin (Figure 7d,e). However, the surface of the fracture seemed unremarkable and smooth, which indicated that the fibers did not slip at the fractured surface (Figure 7f,g). Due to the improved compatibility between the cellulose and the matrix, the tensile strength increased from 33.7 to 41.8 MPa, and the Young’s modulus increased to more than 70%.

Furthermore, NC hydrogels and aerogels can also be modified by solution-immersion processing. For instance, it was reported that CNF aerogels were modified by triethoxyl(octyl) silane via chemical vapor deposition (CVD) to increase hydrophobicity and oleophilicity [99]. The ultra-light CNF aerogels were supported by a dandelion, and the silane-modified CNF aerogels were completely non-wettable by water (Figure 7h,i). The as-fabricated aerogels with improved wet mechanical properties absorbed 210 times more water and 375 times more chloroform, respectively. In addition, the aerogel apparently absorbed chloroform from the bottom of the water (Figure 7j). Except for films and aerogels, CVD was used to modify CNF filaments with organosilanes (Figure 7k) [100]. As shown in Figure 7l, after TC-treated modification, the surfaces of the filaments presented hairy features with lengths ranging from 30 to 40 μm, whereas the DC exhibited continuous homogeneous coating layers (Figure 7m). After the modification, the wet strength and Young’s modulus of the modified filament increased to 160 MPa and 10 GPa, respectively.

### 3.6. Surface Coating and Adsorption of NC

**Figure 8:**
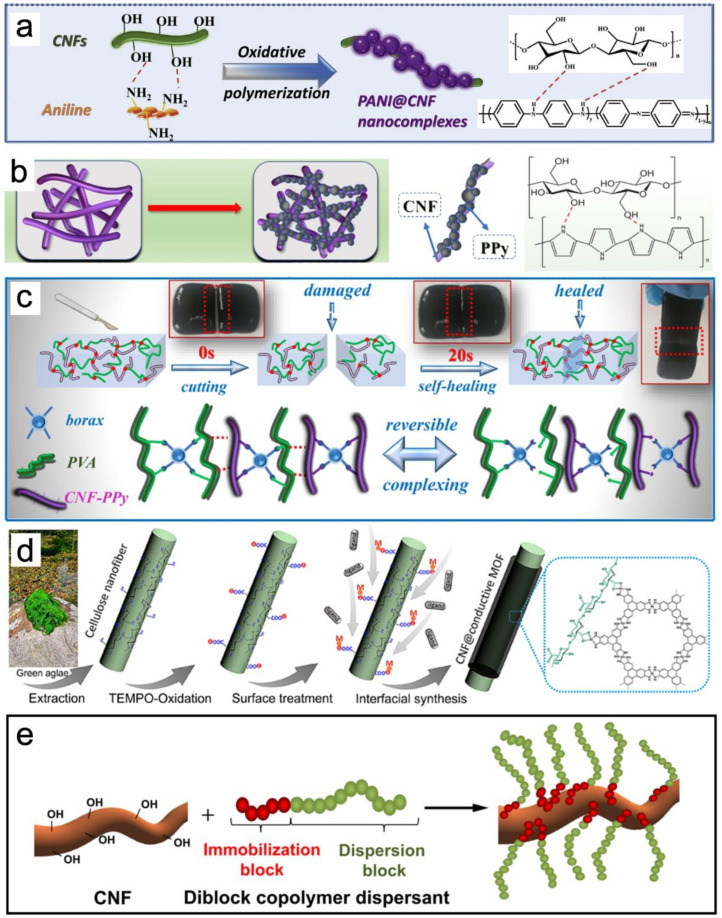
Schematic illustration of the formation of CNF-PANI complexes (**a**) [101]; demonstration of the construction mechanism of PPy-CNF nanocomplexes (**b**) [102]; schematic illustration of in situ self-healing and dynamic reversible cross-links of hydrogels (**c**) [102]; schematic of synthesis procedure for TOCNF@MOFs hybrid nanofibers (**d**) [103]; schematic illustration of adsorption of polymer dispersant onto CNF (**e**) [106].

Generally, the surface coating and adsorption of NCs are employed in packaging, wearable sensors, flexible electrodes, and paper coating. In particular, conducting polymer hydrogels, which offer biocompatibility, viscoelasticity, and good mechanical performance, are considered smart and soft solutions for various advanced applications. For instance, conductive polymers, such as polyaniline (PANI) and polypyrrole (PPy), can be coated on the surfaces of CNFs to obtain conductive functional materials (Figure 8a,b). Because of the interaction between the hydroxyl groups on the CNFs and amine groups of aniline (ANI) monomers or N-H in the pyrrole (Py) ring, PANI or PPy continued to deposit on the surface of the CNF through hydrogen bonding to form conductive nanocomposites [101]. The resultant CNF-PANI exhibited enhanced tensile strength (9.7 MPa), a high Young’s modulus (10.9 MPa), and excellent conductivity (8.95 × 10^−1^ S/m). The material therefore has great potential for use in flexible electrodes, sensors, and paper-based devices. Furthermore, CNF-PPy was further dispersed into the polyvinyl alcohol and borax matrix (PB) to prepare CNF-PPy/PB hybrid hydrogels [102]. The obtained CNF-PPy/PB exhibited enhanced compression stress (22 MPa), low density (1.2 g/cm^3^), and excellent conductivity (3.65 ± 0.08 S/m), respectively. As shown in Figure 8c, the surfaces of the hydrogels adhered to each other, and completely self-healed within 15–20 s. This phenomenon was caused by the dynamically reversible crosslinks formed through hydroxyl groups and borax multi-complexation. In addition to PNI and PPy coating, metal–organic frameworks (MOFs) were coated on the surface of TOCNF to form TOCNF@MOF hybrid nanofibers (Figure 8d) [103]. An aqueous solution containing Ni(OAc)_2_·4H_2_O and 2,3,6,7,10,11-hexahydroxytriphenylene (HHTP) (or 2,3,6,7,10,11-hexaaminotriphenylene (HITP)) was added to exchange ions for the preparation of Ni-HHTP or Ni-HITP. Next, TOCNF@MOFs nanopapers were prepared through the vacuum filtration of the homogeneous suspension, which can be used in flexible energy-storage devices. An excellent electromagnetic-interference-shielding material was fabricated using CNF, MXene, and FeCo by using the layer-by-layer vacuum-filtration method [104]. The produced CNF@MXene@FeCo film possessed remarkable electromagnetic interference (EMI) shielding effectiveness (SE) (58.0 dB) and a low reflection coefficient (0.61). Recently, PANI was dropped on cellulose paper to fabricate a highly flexible, stable, and sensitive sensor (response (≤220 s)/recovery (≤150 s)) with disposable humidity (1.1701 Ω/%RH) [105]. In addition, due to the presence of van der Waals interactions, hydrogen bonds, ionic interactions, and other affinities, physical adsorption is another way to modify CNF. A diblock copolymer dispersant, poly(lauryl methacrylate)-b-poly(2-hydroxyethyl methacrylate) (PLMA-b-PHEMA), was absorbed into cellulose to improve the dispersion of hydrophilic CNF in the hydrophobic polyethylene matrix (Figure 8e) [106]. The contact angle of the modified CNF increased from 48 to 101°, and the Young’s modulus and tensile strength increased by more than 140% and 84%, respectively. In conclusion, the surface modification of CNF using adsorption or coating is an easy way to fabricate conducting polymers, whereas, without grafting, there is a risk of migration or leaching phenomena.

### 3.7. Grafting Modification of NC

As mentioned above, the chemical grafting modification of NC is usually performed by using esterification, etherification, cationization or silanization, in which single molecules react with the hydroxyl groups of NC. Furthermore, the grafting of polymers onto NC can be performed by using the “grafting from” or “grafting onto” methods. The “grafting from” strategy consists of mixing the NC with a monomer and an initiator. Next, polymerization occurs at the surface of the NC, while some non-grafted polymers usually remain in the solution. In the second strategy, NC is mixed with a polymer in a low grafting density and a coupling agent drives the grafting.

**Figure 9:**
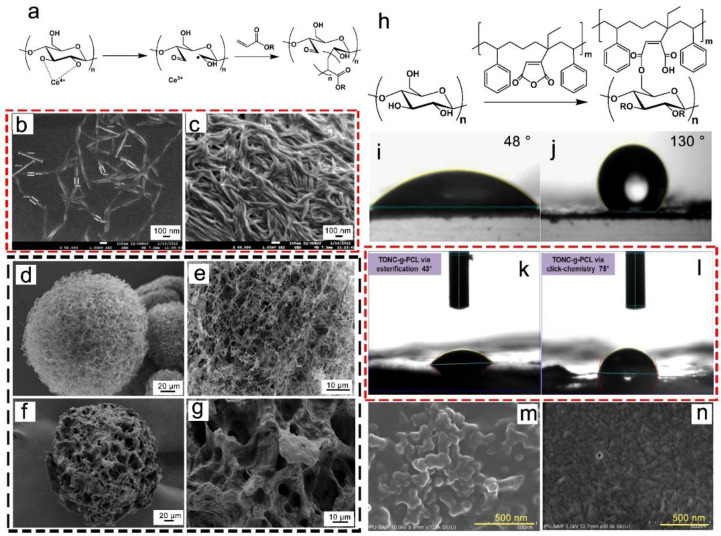
Mechanism of cerium-initiated copolymerization (**a**) [107]; SEM images of CNF (**b**) and CNF/PANI (**c**) [108]; SEM images of CNF cryogel microspheres (**d**,**e**) and CNF-NIPAm hybrid microspheres (**f**,**g**) [109]; mechanism of maleated-styrene-block-copolymer-grafted CNF (**h**) [110]; contact-angle images of CNF (**i**) and maleated-styrene-grafted CNF (**j**) [110]; contact-angle images of polycaprolactone-diol-grafted TOCNF esterification (**k**) and click-chemistry (**l**) [111]; SEM images of DANC/chitosan composite (**m**) and film (**n**) [112].

It was reported that CNF was grafted from various acrylic monomers (butyl acrylate, glycidyl methacrylate, methyl methacrylate, ethyl acrylate, and 2-hydroxyethyl methacrylate) via a redox-initiated free-radical method, with cerium ammonium nitrate used as the initiator [107]. The mechanism of cerium-initiated copolymerization is shown in Figure 9a. All the modifications made the CNF more hydrophobic, and the structure of the CNF was retained and surrounded by a thin coating. Moreover, the CNF was also coated with polyaniline (PANI) through the in situ polymerization of aniline to produce CNF-PANI (Figure 9b,c), leading to roughness on the CNF surface [108]. Next, nature rubber reinforced with CNF-PANI was obtained. The resultant materials, which have electronically conductive properties, can be used in wearable electronics and pressure sensors. In addition, a temperature-sensitive monomer, N-isopropylacrylamide (NIPAm), can be grafted onto CNF cryogel microspheres for controlled drug release [109]. After coating with NIPAm polymer chains, the CNF-NIPAm hybrid microspheres with uneven pore sizes were significantly different from the CNF cryogel microspheres with highly porous and homogeneous network structures (Figure 9d–g).

The “grafting onto” strategy has been used to limit fibril aggregation, reduce cellulose hydrophilicity and modify CNF surfaces. It was reported that maleated-styrene-block copolymers were grafted onto the surface of a CNF, increasing the CNF’s thermal stability and decreasing its crystallinity (Figure 9h) [110]. Moreover, they have also been used to produce hydrophobic CNF with a contact angle of 130°, which was mixed with a poly(styrene) matrix to improve the mechanical properties of the final composites (Figure 9i,j). In order to ensure its hydrophobicity and improve the dispersion, polycaprolactone–diol was grafted onto TOCNF using two different strategies: click-chemistry and esterification [111]. The click-chemistry method led to the strong hydrophobization of the obtained material (contact angel 75°), while the esterification failed to produce hydrophobic materials (the contact angle was only 43°) (Figure 9k,l). Furthermore, DANC obtained via NaIO_4_ oxidation was further grafted with the amino groups of chitosan to form a Schiff base [112]. The as-fabricated DANC/chitosan was casted into films with excellent antimicrobial properties against *Escherichia coli* and *Staphylococcus aureus*, indicating that chitosan-grafted DANC can be used for antimicrobial packaging (Figure 9m,n).

## 4. Summary and Outlook

In summary, various modification methods for obtaining NCs with different functionalities are reported, and novel modification methods are further developed. These obtained NCs can be used as advanced materials to prepare films, wearable sensors, cellulose nanospheres, aerogels, hydrogels, and nanocomposites. However, due to the high energy consumption, the usage of toxic chemical reactants, the high consumption of water, the difficulties involved in scale up, and the uniformity in NC manufacturing, it remains challenging to achieve the sustainable and scalable production of NCs with high final quality and high reactivity. Additionally, more research and investigations should be conducted on the development of efficient pretreatment methods for NC production from an environmental point of view to decrease energy consumption. So far, some pretreatments have been applied to swell cellulose fibers and loosen interfibrillar hydrogen bonds, such as TEMPO-mediated oxidation, ILs, enzymatic hydrolysis, DES, molten-salt hydrates, and others, which support the subsequent NC production and NC functionalization. It should be pointed that the high cost of NCs is the major barrier to its industrial-scale production. Therefore, reductions in the cost of producing functionalized NCs are required. They might be achieved by using low-cost starting cellulosic materials (e.g., agricultural waste, corncob residue derived from xylan plants, sugarcane bagasse from sugar plants, or recycled fibers of wastepaper), developing greener and more efficient approaches to NC production, and better control over preparation process.

On the other hand, some drawbacks limit the use of NCs, such as their low concentration, aggregation, and compatibility with hydrophobic matrices, which can be addressed by the chemical modification of NC. Periodate and NHPI oxidation can be used to control the degree of oxidation and maintain the backbone of cellulose, and the formation of reactive groups (such as carboxylic or dialdehyde groups) on the surfaces of NC can be further modified or reacted with other functional groups. Generally, esterification and etherification are widely used, and the degree of substitution of esterification (0.06–1.5) is relatively higher than that of etherification (0.1–0.78). Further attention should be focused on the development of simple, low-cost, and innovative routes to ensure the efficient scaling up and uniformity of NC manufacturing. The silanization of NC is very important to increase its hydrophobic properties. Furthermore, surface modifications of NCs, such as coating, adsorption, and grafting, are used to provide various functional groups for different end-use purposes, such as antimicrobial applications, electrically conductive devices, or pollutant absorption. However, maintaining the natural morphology of NC, preserving its native crystalline structure, and preventing polymorphic changes in NC are even more challenging during chemical modification. Therefore, further attention should be focused on the development of innovative and high-efficiency chemical routes to ensure the efficient scaling up and uniformity of NC manufacturing, with high end-product quality. Furthermore, most of these functionalization methods for NC production are not environmentally friendly; thus, greater effort is needed to investigate the sustainable and green production of NC. So far, various NC-based materials have been used in high-performance functional materials. The impact of the functionality of the NC on the application performance of the corresponding NC-based functional material should be comprehensively investigated to gain a deeper and better understanding of the structure–activity–application relationships and encourage the practical utilization of these materials. Undoubtedly, with further efforts and updates in the future, it can be predicted that functionalized NC and NC-based products will become much more widely available for practical applications.

## Data Availability

Not applicable.
